# van der Waals Decoration of Ultra-High-*Q* Silica Microcavities for χ^(2)^–χ^(3)^ Hybrid Nonlinear Photonics

**DOI:** 10.1021/acs.nanolett.4c00273

**Published:** 2024-04-01

**Authors:** Shun Fujii, Nan Fang, Daiki Yamashita, Daichi Kozawa, Chee Fai Fong, Yuichiro K. Kato

**Affiliations:** † Quantum Optoelectronics Research Team, RIKEN Center for Advanced Photonics, Saitama 351-0198, Japan; ‡ Department of Physics, Faculty of Science and Technology, Keio University, Yokohama 223-8522, Japan; § Nanoscale Quantum Photonics Laboratory, RIKEN Cluster for Pioneering Research, Saitama 351-0198, Japan; ∥ Platform Photonics Research Center, National Institute of Advanced Industrial Science and Technology (AIST), Ibaraki 305-8568, Japan; ⊥ Research Center for Materials Nanoarchitectonics, National Institute for Materials Science, Ibaraki 305-0044, Japan

**Keywords:** two-dimensional materials, ultra-high-*Q* microcavities, second-harmonic generation, nonlinear optics, transition metal dichalcogenides

## Abstract

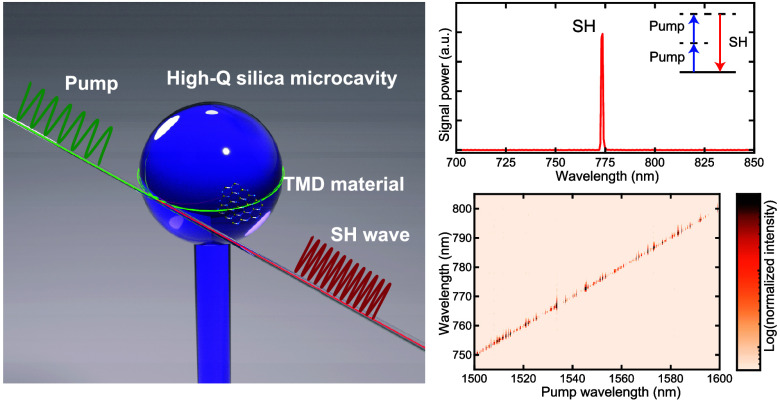

Optical nonlinear processes are indispensable in a wide range of applications, including ultrafast lasers, microscopy, and quantum information technologies. Among the diverse nonlinear processes, second-order effects usually overwhelm the higher-order ones, except in centrosymmetric systems, where the second-order susceptibility vanishes to allow the use of the third-order nonlinearity. Here we demonstrate a hybrid photonic platform whereby the balance between second- and third-order susceptibilities can be tuned flexibly. By decorating ultra-high-*Q* silica microcavities with atomically thin tungsten diselenide, we observe cavity-enhanced second-harmonic generation and sum-frequency generation with continuous-wave excitation at a power level of only a few hundred microwatts. We show that the coexistence of second- and third-order nonlinearities in a single device can be achieved by carefully choosing the size and location of the two-dimensional material. Our approach can be generalized to other types of cavities, unlocking the potential of hybrid systems with controlled nonlinear susceptibilities for novel applications.

Since the landmark discovery of second-harmonic generation (SHG)^1^ enabled by the invention of lasers,^2^ nonlinear optics have played a central role in the development of diverse photonics applications. Frequency conversion processes are particularly important, being extensively employed in ultrafast optics,^3^ metrology,^4,5^ quantum state generation,^6,7^ and microscopy.^8,9^ To achieve these functionalities, both second- and third-order processes, such as SHG, third-harmonic generation (THG), sum-frequency generation (SFG), parametric downconversion, and four-wave mixing (FWM), are utilized.

With such a variety of nonlinear effects, combinations of frequency conversion processes would allow for a more flexible spectral synthesis. The efficiencies of nonlinear processes depend directly on the nonlinear susceptibility of conversion media, but the origins are markedly different for second- and third-order susceptibilities. An essential requirement for second-order nonlinear processes to occur is inversion symmetry breaking, and typical materials include dielectric crystals (for example, lithium niobate and β-barium borate), III–V semiconductors, and organic crystals.^10^ Although second- and third-order nonlinearities can coexist in nanoscale structures such as dielectric nanoparticles,^11^ nanocrystals,^12^ and layered nanomaterials,^13,14^ conventional nonlinear optical materials with broken inversion symmetry exhibit strong second-order susceptibility that overwhelms other higher-order nonlinearities. Conversely, second-order nonlinear susceptibility vanishes in centrosymmetric crystals and amorphous materials (e.g., liquids, gases, and amorphous solids), and only third-order processes can be utilized in these χ^(3)^ materials.

In this regard, one promising strategy is to establish a hybrid system by combining a noncentrosymmetric nonlinear material with an ultra-high-*Q* microcavity fabricated from a χ^(3)^ material.^15^ The strength of second-order processes can be controlled through mode overlap with the noncentrosymmetric material, while exceptional enhancement of optical density in the tiny mode space can be facilitated to boost the third-order process to a practical level.

As a candidate system, we propose ultra-high-*Q* silica microcavities decorated by transition metal dichalcogenides (TMDs). Silica whispering-gallery microcavities boast ultrahigh *Q* values (>10^8^) that ensure high-circulating optical intensities essential for inducing various optical nonlinear processes^16−23^ with a moderate continuous-wave (CW) excitation. Meanwhile, monolayer TMDs possess a magnitude of second-order nonlinearity comparable to that of commonly used nonlinear crystals^24−26^ and are thus expected to be used for practical nonlinear applications.^27−32^ Their atomically thin nature gives them mechanical flexibility to conform to the surface of the optical microcavities, and the van der Waals character makes them compatible for the heterogeneous interface.^33−36^

Here, we demonstrate a novel nonlinear photonic platform by decorating ultra-high-*Q* silica microspheres with tungsten diselenide (WSe_2_). Atomically thin layers of the two-dimensional (2D) material are transferred onto the cavity with a minimal level of scattering loss. Cavity-enhanced second-harmonic (SH) generation is achieved by CW excitation with only a few hundreds of microwatts because of the strong light–matter interactions between a resonant optical field and integrated WSe_2_. We also observe efficient SFG with a two-color excitation scheme. In addition, the pump power dependence shows self-locking of the SH output, revealing the mechanism of the dynamic phase-matching process. It is confirmed that the SH process occurs for only odd layer numbers, and the coexistence of second- and third-order nonlinearities in a single device is achieved by controlling the second-order susceptibility of the device.

Figure 1a shows a conceptual illustration of a 2D material-decorated silica microcavity, capable of serving as a second-order nonlinear photonic platform. Strong light–matter interaction assisted by cavity resonance permits efficient nonlinear optical processes that originate from the atomically thin layered material with low-power CW excitation. The frequency-converted light that resonates with another longitudinal resonance mode, in a situation termed a doubly resonant condition, allows the cavity-enhanced signals to couple to the same waveguide coupler utilized for excitation. The normalized mode intensity of a microsphere cavity is shown in Figure 1b, where the inset shows the optical mode profile (the relationship between the evanescent field ratio and the cavity radius is further detailed in the Supporting Information).

**Figure 1:**
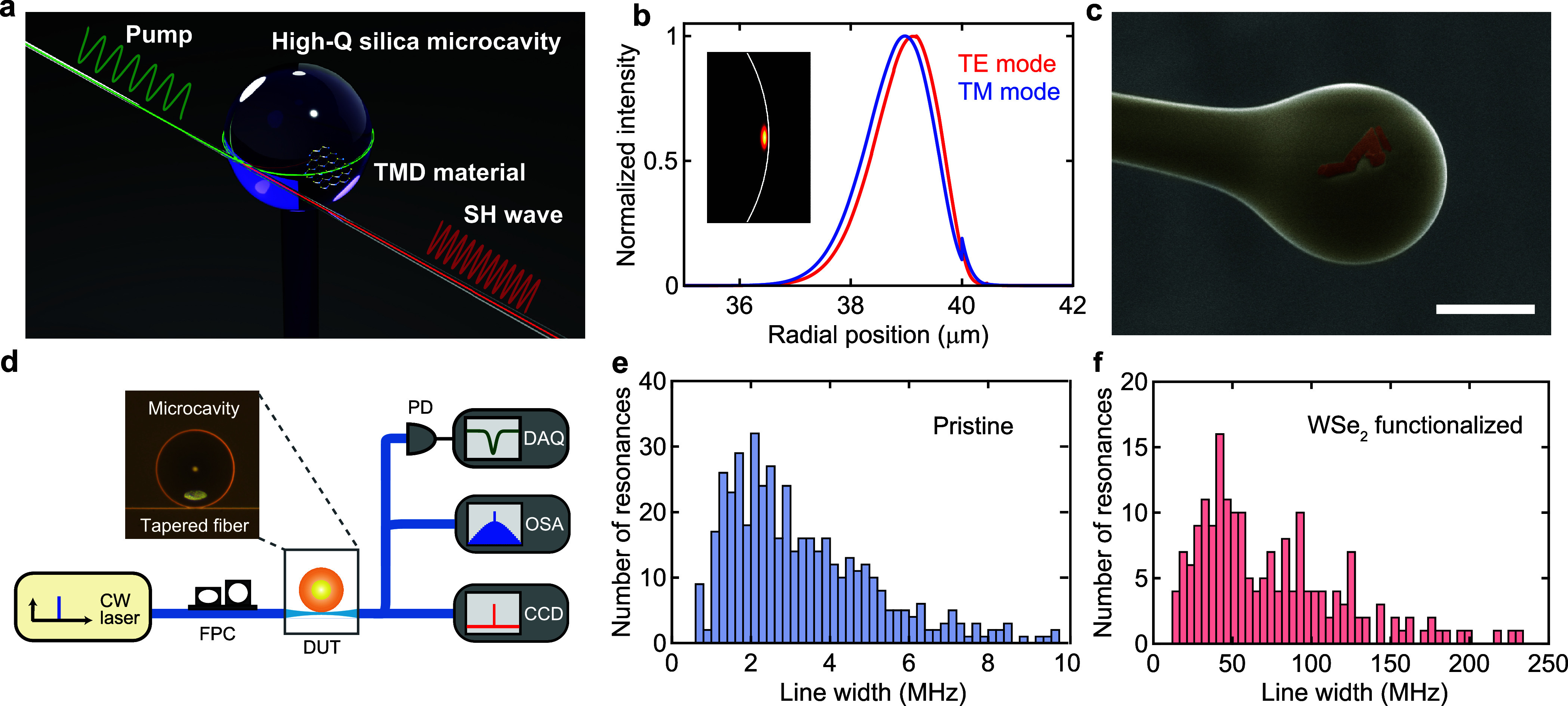
van der Waals decoration of a high-*Q* silica microcavity by atomically thin 2D material. (a) Conceptual illustration of a monolayer material-integrated silica microcavity realizing strong light–matter interaction. (b) Simulated normalized intensity of the optical mode across the equator of a silica microsphere with a radius of 40 μm. The calculations are conducted by using finite element method (FEM) software (COMSOL Multiphysics). The cavity modes exhibit a slight difference in the profiles, and transverse-magnetic (TM) modes exhibit evanescent fields slightly higher than transverse-electric (TE) modes. The inset shows the optical mode profile, where the white line indicates the boundary between the silica and surrounding air. (c) False-color scanning electron micrograph image of a WSe_2_-integrated high-*Q* microsphere. The image has been retouched to show WSe_2_ in red and the microsphere in yellow. The scale bar is 50 μm. (d) Experimental setup. Abbreviations: FPC, fiber polarization controller; DUT, device under test; PD, photodetector; DAQ, data acquisition; OSA, optical spectrum analyzer; CCD, charged-coupled device installed in a spectrometer. (e and f) Histograms of cavity line widths in pristine and decorated microcavities, respectively. The degradation of *Q* factors is mainly attributed to an increase in surface scattering loss.

We first decorate a silica microsphere cavity (diameter of ∼80 μm) by transferring mechanically exfoliated monolayer WSe_2_ onto the cavity surface using the polydimethylsiloxane (PDMS)-assisted dry-transfer technique.^37^ The layer numbers of WSe_2_ flakes are identified either through photoluminescence (PL) measurement^38^ or by optical contrast in microscope images prior to the transfer.^39^Figure 1c shows a false-color image of the WSe_2_-decorated silica microsphere cavity (details of sample fabrication and interaction length presented in the Supporting Information).

To characterize the influence of the WSe_2_ flake on the *Q* factor of a microcavity, we compare the transmission spectra before and after the transfer process. The experimental setup is presented in Figure 1d. All resonances observed within the range of 1530–1570 nm are numerically fitted to a Lorentzian function. This allows for the statistical analysis of the loaded full width at half-maximum line width (=ω/*Q*) as shown in panels e and f of Figure 1. The median value in a pristine (i.e., before transfer) microsphere is 2 MHz, which corresponds to an ultrahigh *Q* factor of 1 × 10^8^. After the transfer of a WSe_2_ flake, the most probable loaded line width broadens to approximately 40 MHz, corresponding to a *Q* factor of 5 × 10^6^ even though the highest *Q* values are ∼10^7^.

The degradation in the *Q* factor is likely due to an increase in scattering loss resulting from the decoration, which is also observed in the integration of materials into other nanophotonic cavities.^33,40,41^ We anticipate a minimal effect on the *Q* factor from the absorption loss caused by the WSe_2_ flake because the telecom band photon energy is significantly lower than the bandgap of monolayer WSe_2_ (∼1.75 eV). For the same reason, we do not expect significant damage to the 2D material. It should be noted that the uniformity of transferred flakes is the key to maintaining high *Q* factors as well as the flake size and the transferred position, and placing a small flake away from the equator of a microcavity would greatly reduce the scattering loss in high-*Q* modes. For most of this study, however, we place priority on using uniform and large WSe_2_ flakes and transfer onto the equator of the device to maximize the interaction length between the optical modes and the WSe_2_ material.

Figure 2 presents optical spectra in the visible and corresponding pump wavelength bands. By carefully tuning the pump laser wavelength to a cavity resonance with a pump power of 500 μW, we clearly observe second-harmonic (SH) light (Figure 2a,b). The frequency of the SH light (773.1 nm) exactly matches twice the pump frequency (1545.5 nm) with a wavelength error of only 0.045%, and this fact confirms the occurrence of a frequency-doubling process via second-order optical nonlinearity. We stress that other third-order (Kerr) nonlinear processes, which could arise from bulk silica microcavities, are absent in this experiment, because the threshold powers are far beyond our pump power level. The required pump powers for FWM and Raman oscillation are 12.6 and 36.1 mW, respectively, in the case of a loaded *Q* factor of 5 × 10^6^, as threshold powers of these processes scale as *V*/*Q*^2^.^17,18^

**Figure 2:**
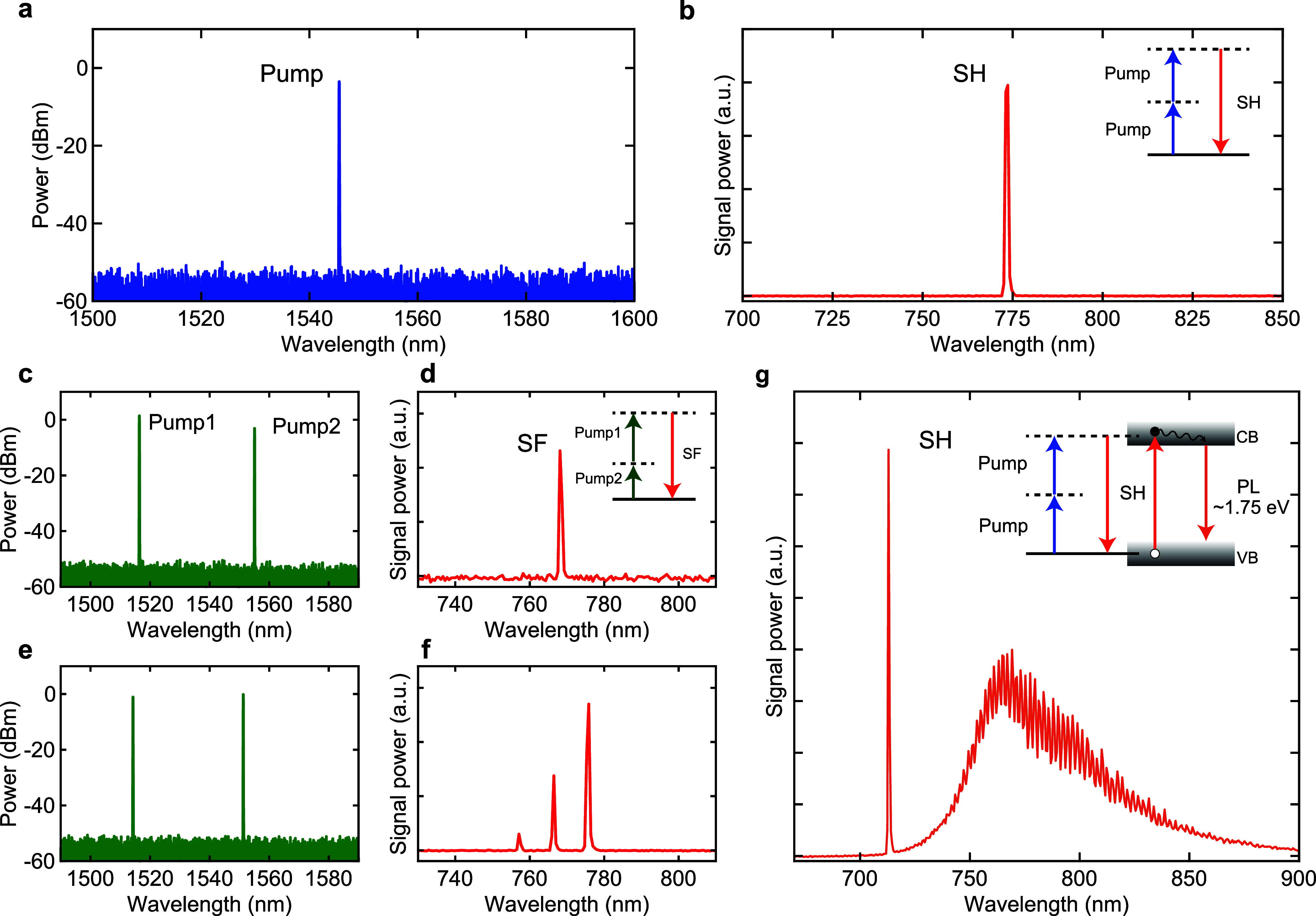
Observation of second-order nonlinear processes in material integrated microresonators. (a and b) Optical spectra of pump wavelength and generated SH light. The frequency of the SH light exactly matches twice the frequency of the pump light, indicating the nonlinear frequency-doubling process. The energy diagram of an SH process is shown in the inset. (c and d) Optical spectra of two different pump wavelengths and generated second-order sum-frequency (SF) light. The frequency of SF light corresponds to the sum of pump frequencies as depicted in the inset. (e and f) Measured spectra of pump wavelengths and corresponding visible light, where the two-color excitation scheme enables simultaneous generation of SFG and SHG. The difference in signal powers is due to the phase-matching condition for near-infrared and visible cavity modes. (g) Optical spectra of SHG-mediated photoluminescence (PL) emission in a WSe_2_-decorated cavity. The SH light at a wavelength of 715 nm excites excitonic PL in a monolayer WSe_2_, where the broad emission is optically coupled to numerous cavity modes. The inset shows the energy diagram of the process. Abbreviations: CB, conduction band; VB, valence band.

Next, we pump the device by using two CW lasers with different frequencies (i.e., two-color excitation) at submilliwatt pump powers. This scheme allows us to observe SFG as shown in panels c and d of Figure 2. A two-color pump imposes a triply resonant condition on the sum-frequency process to be phase-matched, but it is easy to find the phase-matching condition by slowly tuning one laser while keeping the frequency of the other laser within a high-*Q* resonance. Panels e and f of Figure 2 show a unique example, where two SH and one SF light are generated from two laser inputs because of 5-fold resonant triple-phase matching.

In addition to second-order nonlinearities, we also observed excitonic photoluminescence (PL) from the monolayer WSe_2_. Figure 2g shows a spectrum of SHG at a wavelength of 715 nm and the associated PL emission when the device is pumped at a wavelength of 1530 nm. The multiple spikes seen in the PL spectrum indicate that broad excitonic PL couples to the high-*Q* cavity modes and the intensities are enhanced due to the Purcell effect or modulated by the differing collection efficiencies. The energy diagram is depicted in the inset of Figure 2g. We emphasize that the observation of this unique resonance energy transfer, i.e, SHG-mediated PL and subsequent resonant enhancement, has become possible only with our WSe_2_-decorated high-*Q* devices. We note that there is a possibility of two-photon absorption PL simultaneously occurring under the infrared excitation,^42^ while it is difficult to distinguish these processes from optical spectra. This result also proves the strong interaction between a monolayer WSe_2_ and whispering-gallery modes via an evanescent field.

The dynamic phase matching is highlighted in the pump power dependence of the SH power, as shown in Figure 3a (see the Supporting Information for details of the dynamic phase-matching process). We measure the SH power for the same cavity mode and carefully tune the pump wavelength so that the SH light is maximized at each pump power. This measurement scheme allows us to find the perfect phase-matching condition at a certain pump power, which can be dynamically altered by the nonlinear resonance shifts. The double logarithm plot is presented in the inset of Figure 3a, where three distinct regimes can be recognized. Below a pump power of ∼2 mW, the SH powers exhibit a linear slope of ∼2.2, which is very close to the anticipated slope of 2 for an SHG process. As the pump power is increased from 2 to 4.5 mW, the fitted slope drastically changes to ∼5.5, and a further increase in the pump power (>4.5 mW) induces saturation of the SH power. Such a kink behavior of the SHG intensity has not been reported in conventional SHG measurements of TMD flakes on substrates^43,44^ or photonic nanostructures.^31,41,45,46^

**Figure 3:**
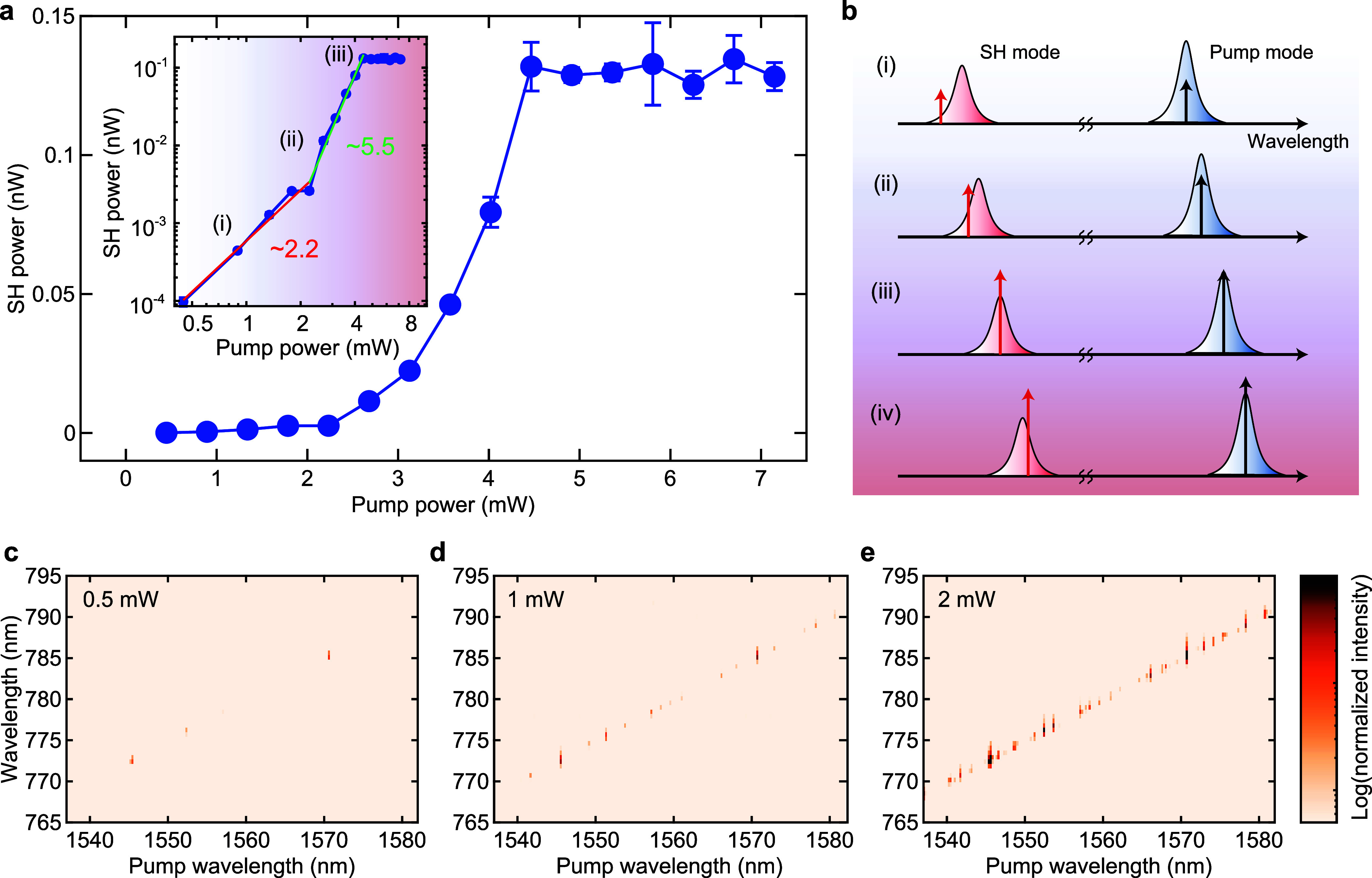
SH power dependence on the pump power. (a) Maximum SH power as a function of pump power for the same cavity mode. The data are presented in a log–log scaling with power-law fits in the inset. The SH powers exhibit a slope of ∼2.2 at the relatively low pump power regime (<2 mW), but the slope increases to ∼5.5 in the intermediate region (2–4.5 mW). In the high-pump power region (>4.5 mW), the SH power starts to saturate and remains stable. The error bars correspond to the standard deviation of 10 repeated measurements. (b) Schematic for the mechanism of the dynamic phase-matching process. (c–e) Spectral mapping for different pump powers. The SH power exhibits a significant dependence on pump powers of 0.5, 1, and 2 mW, respectively. The color maps are normalized to a common scale.

We therefore consider the influence of the dynamic phase-matching condition in a double-resonance system. Figure 3b shows the schematic for the mechanism under consideration, where the SH light is blue-detuned at low pump powers. In this scenario, SH light is considered to be almost in an off-resonance condition with a large detuning [state (i)], yielding a moderate conversion efficiency with a slope of ∼2. As the pump power increases, thermal and Kerr nonlinearities induce a significant red-shift of the resonances.^47^ While the frequency of the SH light is twice the pump frequency (i.e., ω_SH_ = 2ω_p_), resonance mode ω_2_ for SH generally shows shifts smaller than those of SH light (Δω_2_ < Δω_SH_) due to the imperfect mode overlap between the pump and SH modes.^48^ The detuning of the SH light therefore decreases at a higher pump power, leading to a rapid increase in conversion efficiency [state (ii)]. Once the SH power reaches its maximum when both cavity modes exactly match the on-resonance condition [state (iii)], a further increase in intracavity power results in the red detuning of SH light, which would reduce the output [state (iv)]. The maximum SH power for higher pump powers would then be obtained for the specific intracavity power, where the double-resonance condition is retained. Because the intracavity power is almost constant, the SH power saturates despite a further increase in pump power. Such a complex power dependency is clearly observed in a separate experiment, where we record SH signals while continuously scanning the pump laser frequency at a certain pump power. As shown in Figure 3c–e, the SH signal becomes more and more frequent in the spectral map, and the intensity is drastically enhanced with an increase in pump power. Given the presence of numerous higher-order modes in silica microspheres, the existence of fundamental and SH modes that fulfill the phase-matching condition is plausible for different order modes.^16,20^ We note that no pump polarization dependence is observed (extended data presented in the Supporting Information).

It is possible to calculate the conversion efficiency from the data depicted in Figure 3a. When we define *P*_SH_ as the detected SH power, the calculated maximum conversion efficiency (*P*_SH_/*P*_p_^2^) is 6.6 × 10^–4^ % W^–1^ with a pump power *P*_p_ of 4.5 mW. It should be noted that the internal (intracavity) conversion efficiency is expected to be >1 order of magnitude higher than the value presented above because the waist of the nanofiber waveguide is optimized to the pump wavelength band in this experiment, thus resulting in the poor coupling efficiency of SH light due to the phase mismatch between the visible band and the nanofiber coupler.^49,50^ We note that the collection efficiency can be improved by employing an additional nanofiber designed for SH wavelengths, i.e., add–drop configuration,^22,48^ or by exploiting a chaotic channel in deformed microcavities.^50^

As mentioned above, symmetry plays an important role in determining the nonlinear susceptibility, and therefore, the number of layers in the two-dimensional material is a crucial factor. The WSe_2_ crystals used in this work possess the 2H-phase (semiconducting) structure, which is more stable than other crystal phases. The 2H-phase TMD crystals belonging to space group *D*_3*h*_ exhibit substantial second-order nonlinearity for only odd layer numbers, whereas the χ^(2)^ nonlinearity vanishes in even layer numbers because the net nonlinear dipoles are canceled out due to inversion symmetry.^43,44^ Considering these selection rules, we performed a comparative experiment in four different devices.

Figure 4 shows the mapping of SH spectra in the visible wavelength region when the pump wavelength is scanned from 1500 to 1600 nm with a pump power of 3 mW. As we anticipate, strong SH light appears only in the ML and 3L-WSe_2_ devices (Figure 4b,d), whereas there is no distinct signal in the pristine and 2L-WSe_2_ devices (Figure 4a,c). This is clear evidence that second-order nonlinearity originates from the integrated WSe_2_, not from intrinsic surface symmetry breaking of the cavity material.^48^ In a pristine device, third-order processes such as THG and third-order SFG associated with pump, FWM, and stimulated Raman scattering (SRS) are observed in the range of 500–620 nm (Figure 4a, left) because of the unaltered ultrahigh *Q* factors (>5 × 10^7^). We find that the number of SH signal peaks in the map is surprisingly high in both ML- and 3L-WSe_2_ devices even though the *Q* factors of most resonances are not as high as 10^7^. It should be noted that the size of the flakes (i.e., interaction length) is approximately the same for each experiment. We attribute the efficient, highly populated SHG to giant second-order nonlinearity of TMD materials and relaxed resonant phase-matching condition due to cavity line width broadening. If we could achieve much higher *Q* factors with a larger overlap between the cavity mode and the material, the conversion efficiency is expected to substantially increase; nevertheless, the resonant phase-matching condition would become stricter as a trade-off.

**Figure 4:**
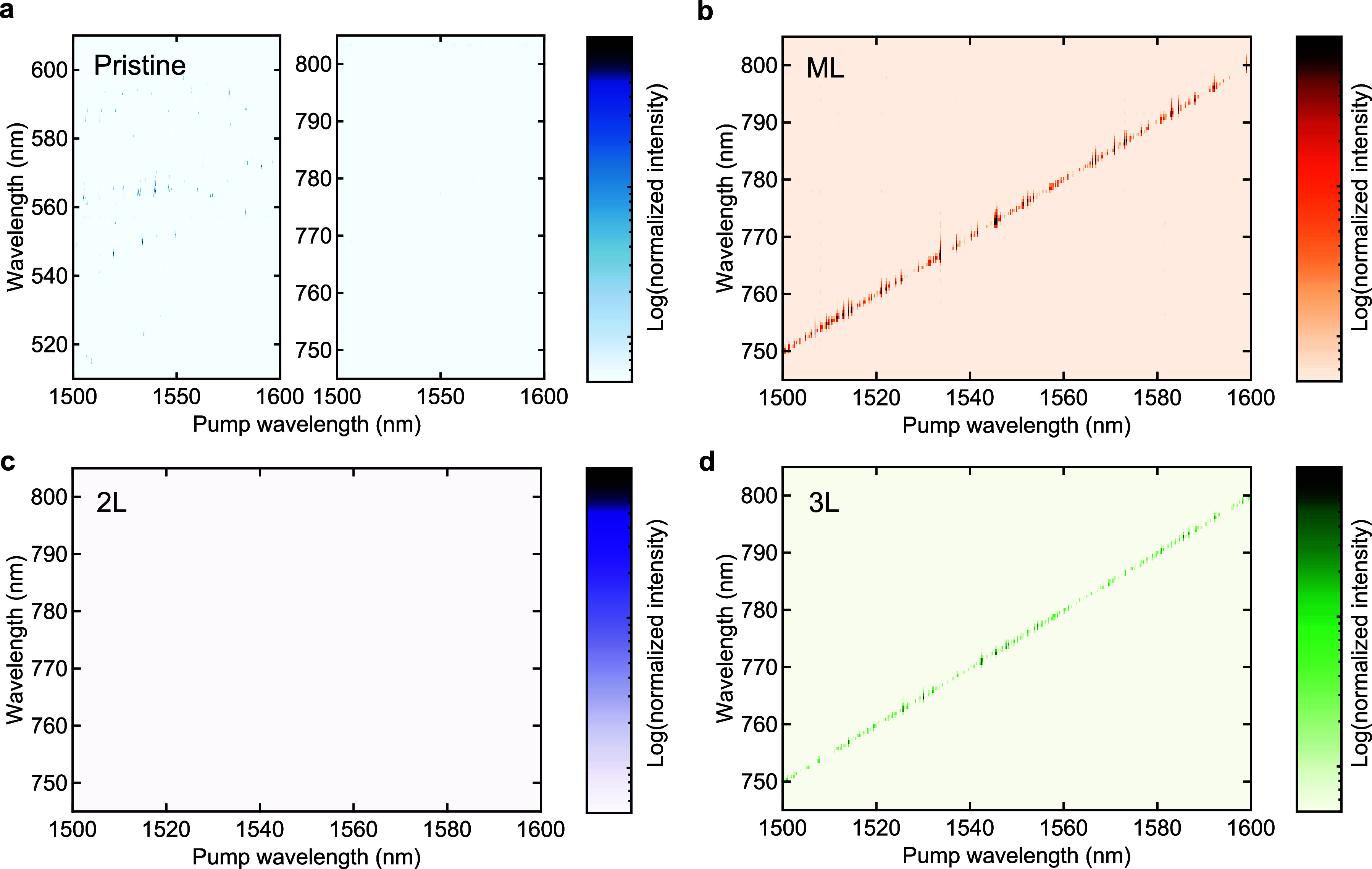
Layer dependence of SH light intensity. (a) Spectral mapping of the signal intensity in the visible wavelength region in a pristine silica microsphere. Third-order nonlinear processes (e.g., THG and TSFG) are observed in the wavelength range of 500–600 nm, whereas no strong signal appears in the wavelength range of 740–810 nm due to the absence of the second-order nonlinearity. (b) Spectral mapping in a monolayer WSe_2_-decorated microcavity. The strong SH signals are observed over a wide range of pump wavelengths (1500–1600 nm). (c) Mapping in a 2L-WSe_2_-decorated sample. No clear SH light is measured in the map because the inversion symmetry exists in a 2H-stacked bilayer WSe_2_; thus, the χ^(2)^ nonlinearity vanishes. (d) Mapping in a 3L-WSe_2_ device. The distinct SH light is observed again similar to the monolayer case because the inversion symmetry is broken for 3L-WSe_2_ crystal structures. The measurements are performed with a fixed pump power of 3 mW.

We have shown thus far the results focused on the emergence of second-order nonlinearity, but one key advantage of this technique is its flexible controllability of nonlinear susceptibility. By carefully controlling the transferred position and the flake size of materials, we can tune the balance between second- and third-order nonlinearity. Here, we intentionally place a small flake (width of <10 μm) away from the equator of a cavity to keep the *Q* factors high enough (>10^7^) to simultaneously observe both second- and third-order nonlinear processes in the same device. A flake position a few micrometers (corresponding to the scale of the cavity mode profile) from the equator balances the *Q* factors and efficient interaction with cavity modes.

Panels a and b of Figure 5 show the observed optical spectra in the pump and the visible wavelength bands in this WSe_2_-decorated microcavity. In the pump wavelength band, FWM sidebands are observed in the vicinity of the pump light and a few Raman peaks can be recognized around 1630–1670 nm, which coincides with the Raman gain band of silica.^18^ For the visible wavelength band, the peaks around 520–600 nm arise from THG and third-order SFG processes involving the peaks seen in the pump band. In particular, the pump and several Raman peaks allow a variety of sum-frequency combinations, resulting in multiple emissions in this regime. The signals around 600 nm are believed to involve a cascaded Raman process.^20,22,23^ While these signals originate from third-order nonlinearity, the strong signal at a wavelength of 772 nm corresponds to the SH light of the pump light via second-order nonlinearity induced by monolayer WSe_2_. The spectral map is shown in Figure 5c, where the strong visible light is recognized as a result of the simultaneous generation of second- and third-order processes. The signals around 780–800 nm come from the second-order SFG process of the pump and Raman components, which are not observed in the previous experiments (Figure 4).

**Figure 5:**
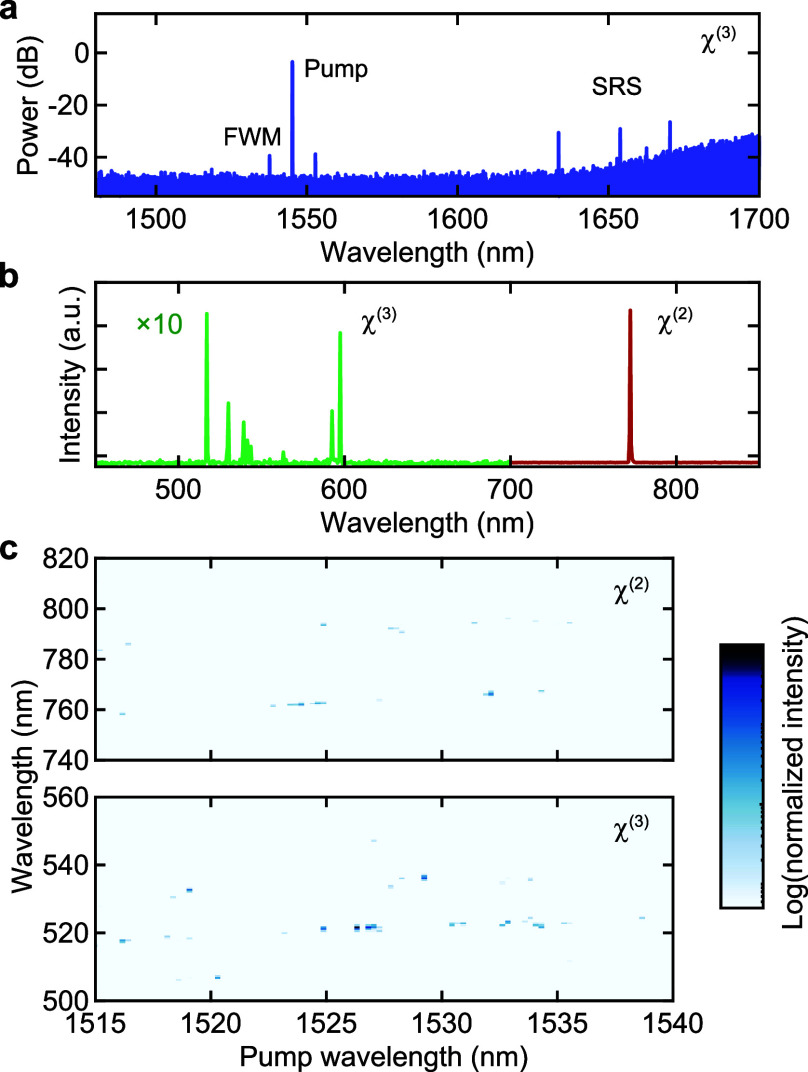
Coexistence of second- and third-order nonlinearities. (a and b) Optical spectra of telecom and visible wavelength regions, respectively. The frequency-converted light is generated via χ^(3)^ nonlinearity in the pump band, resulting in the complex spectrum in the visible band. (c) Spectral mapping of signal intensities in the two different wavelength bands, from 740 to 820 nm and from 500 to 560 nm. The distinct signals are observed in both bands, revealing the coexistence of second- and third-order nonlinearities.

In conclusion, we have demonstrated a novel approach for introducing second-order optical nonlinearity in ultra-high-*Q* silica microcavities through decoration by a two-dimensional material. Via integration of atomically thin TMD layers with broken crystal inversion symmetry onto the surface of amorphous silica microspheres, cavity-enhanced SHG and SFG arise from strong light–matter interactions via evanescent fields. The cavity-enhanced PL emission mediated by the SHG process reveals the distinct optical coupling between SH light and the excitonic resonance of the monolayer WSe_2_. The conversion efficiency of SH light is strongly dependent on the pump power as a result of the dynamic phase-matching process, leading to a drastic increase and saturation of the SH power. A carefully coordinated clean-stamp transfer technique allows for investigation of the layer number dependence, as well as manipulation of the relative strength of the second- and third-order optical nonlinearity in the device.

Practical levels of second-order nonlinearity in χ^(3)^ materials have long been strongly desired. Surface symmetry breaking^48,51^ and photoinduced effects^52,53^ can introduce second-order nonlinear susceptibility but are limited in various aspects. In comparison, this study offers a powerful way to controllably enhance optical nonlinearity in high-*Q* microcavities through the size and placement of the 2D material, which would cause break throughs in nonlinear optics. The results presented in this work lead to an anticipation that optical nonlinearity can be artificially designed in hybrid systems, where various nonlinear processes are combined to implement unconventional functionalities.

In addition, we note that this approach can be extended to other centrosymmetric high-*Q* cavity devices, including integrated ring resonators made of silicon or silicon nitride (Si_3_N_4_), and thus paves the way to few-photon coherent nonlinear optics and quantum photon manipulation in various platforms. The combination of ultra-high-*Q* cavities with nanomaterials opens up a novel regime in the investigation of optical processes at high fields under CW excitation, potentially leading to intriguing physical phenomena as well as nanophotonic applications.

## Supporting Information Available

Simulation of mode profiles, sample fabrication, cavity transmission spectrum, and polarization dependence of the SH intensity (PDF)
