# Supplementary material for: van der Waals Decoration of Ultra-High-Q Silica Microcavities for χ(2)–χ(3) Hybrid Nonlinear Photonics
Source: Nano Lett. 2024 Apr 1;24(14):4209–16. doi: 10.1021/acs.nanolett.4c00273 (PMC11010230; doi:10.1021/acs.nanolett.4c00273)
Supplement: Supplementary file 1 — nl4c00273_si_001.pdf [file nl4c00273_si_001.pdf]

Supporting information to "van der Waals decoration of ultrahigh-Q silica microcavities for $\chi^{(2)}$-$\chi^{(3)}$ hybrid nonlinear photonics"

Shun Fujii,[1,2,*] Nan Fang,[3] Daiki Yamashita,[1,4]
Daichi Kozawa,[1,3,5] Chee Fai Fong,[3] and Yuichiro K. Kato[1,3,†]

[1]*Quantum Optoelectronics Research Team, RIKEN Center for Advanced Photonics, Saitama 351-0198, Japan*

[2]*Department of Physics, Faculty of Science and Technology, Keio University, Yokohama, 223-8522, Japan*

[3]*Nanoscale Quantum Photonics Laboratory, RIKEN Cluster for Pioneering Research, Saitama 351-0198, Japan*

[4]*Platform Photonics Research Center, National Institute of Advanced Industrial Science and Technology (AIST), Ibaraki 305-8568, Japan*

[5]*Research Center for Materials Nanoarchitectonics, National Institute for Materials Science, Ibaraki 305-0044, Japan*

[*] Corresponding author. shun.fujii@phys.keio.ac.jp

[†] Corresponding author. yuichiro.kato@riken.jp

The relation between the evanescent field ratio and the cavity radius

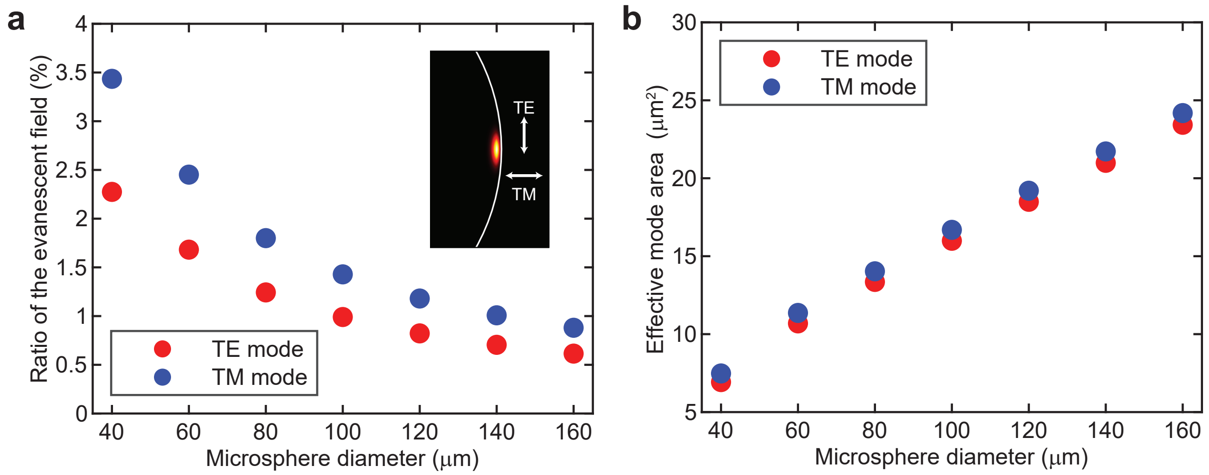

Supplementary Figure S1. The relation between mode profiles and the cavity diameter in a silica microsphere. Calculated mode ratio of the evanescent field to the total field (a) and the cavity mode area (b) as a function of the microsphere diameter for two different polarizations.

The size of a silica microsphere cavity is the key to obtaining strong light-matter interaction between the optical mode and the surface integrated transition metal dichalcogenide (TMD) flake because the ratio of the evanescent field to the total intensity is inversely proportional to the cavity size. Here, the optical mode profiles and the effective mode area are calculated by finite element method (FEM) simulation (COMSOL Multiphysics). Although the optical density is maximized at a few microns inside from the surface of the cavity, evanescent field exists at the boundary, which interacts with a surface integrated 2D material. These simulation results indicate that the evanescent tails, accounting for up to a few percentages of the total light intensity, can interact efficiently with the surface layer for both primary polarization modes, namely the transverse-electric (TE) and transverse-magnetic (TM) modes. The light-matter interaction on the surface layer generally increases in a smaller cavity because the ratio of the evanescent field to the total intensity is inversely related to the cavity radius.

Figure S1(a) shows the calculation result representing the ratio of the evanescent field as a function of the microsphere diameter for two different polarizations (where the TE mode is mainly along the polar direction, whereas the TM mode has two main electric-field components along the radial and azimuthal directions as shown in the inset). This result

indicates that the TM modes exhibit a slightly higher evanescent intensity. It should be noted that the cavity mode volume is proportional to the diameter as shown in Fig. S1(b), in contrast to the evanescent ratio.

Besides, a smaller cavity has another advantage for light-matter interaction because the intracavity power circulating inside the cavity is also inversely proportional to the cavity diameter D as the intracavity power at a critical coupling condition is given by [1],

$$P_{\mathrm{cav}} = \frac{Q f_{\mathrm{FSR}}}{\pi f} P_{\mathrm{in}} = \frac{Qc}{\pi^2 n D f} P_{\mathrm{in}}, \tag{S1}$$

where f_{FSR} is the free-spectral range of the cavity, n is the refractive index, f is the resonant frequency, and P_{in} is the input power.

Device fabrication

A silica microsphere cavity is fabricated from a standard single-mode fiber (SMF-28) via arc-discharging using a commercial fiber fusion splicer. The cavity size can be roughly controlled by the discharge power, position, and duration. The arc-melting process ensures the surface uniformity and smoothness, enabling ultrahigh Q-factors of up to 10^8. The Q-factors in the SH wavelength are not measured in this work, but we expect that the values are similar levels to the telecom wavelength region [2]. Before the cavity fabrication, we reduce the fiber diameter to approximately 20–30 µm by adiabatically stretching the optical fiber. This preprocess allows the fabrication of a microsphere with a diameter of less than 100 µm. Atomically thin WSe_2 flakes are prepared by mechanical exfoliation of a bulk WSe_2 crystal (HQ Graphene). The WSe_2 flake is deposited on the microsphere surface using a dry-transfer technique assisted by a PDMS sheet. In the layer dependence experiment, we select a specific layer of WSe_2 flakes that are as uniform as possible. Precise position alignment is accomplished by using a motorized position system and a high-magnification microscope.

The fabrication process of a 2D-material-decorated microsphere is shown in Fig. S2(a). A device is prepared by the following steps: microsphere fabrication via arc-discharging, positioning, stamping, and releasing. Figure S2(b) and S2(c) show microscope images of the WSe_2 flake before and after the transfer process. Figure S3 shows a typical transmission spectrum of a silica microsphere with a diameter of ∼80 µm. A cavity exhibits many mode families and cavity modes. Conversely, microsphere cavities possess many different modes

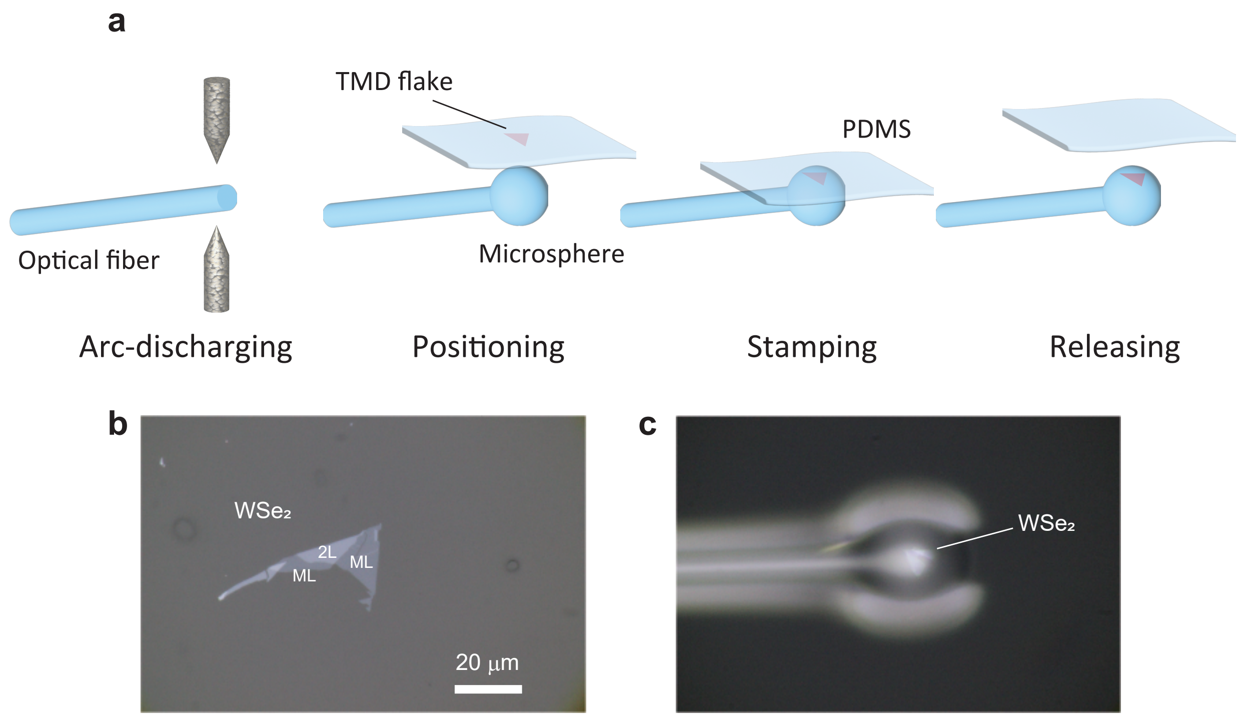

Supplementary Figure S2. Sample fabrication of 2D-material-integrated silica microspheres. (a) Fabrication process. Steps from cavity fabrication to all-dry transfer. (b, c) Microscope images of WSe_2 on a PDMS sheet and functionalized microsphere (image focused on the WSe_2 flake), respectively.

with similar mode distributions and refractive indices, which allows many different modes to interact with the integrated WSe_2.

Experimental setup details

A wavelength-tunable CW laser (Santec, TSL-710) at the telecom band is used as a pump light source. The polarization of the input light is adjusted to the desired cavity modes by using a fiber polarization controller (FPC). A tapered fiber waveguide (diameter of ∼1 µm) is used to couple the pump light to a cavity via evanescent coupling. The distance between the fiber and the cavity is carefully controlled by using a high-precision positioner since the coupling strength is determined by the distance and the coupling position [3, 4]. The pump (telecom band) and visible (400–900 nm) spectra are measured by an optical spectrum analyzer (OSA) and a thermoelectrically-cooled charge coupled device (CCD)

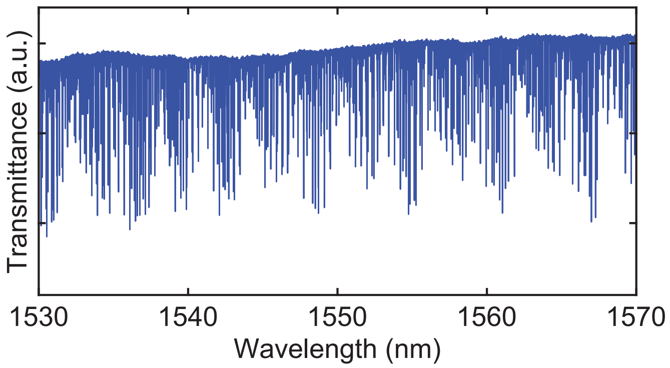

Supplementary Figure S3. Transmission spectrum of a silica microsphere with a diameter of ∼80 µm. Numerous cavity modes are observed within a cavity free-spectral range of ∼6 nm.

camera attached to a high-resolution spectrometer, respectively. The wavelength resolution of the OSA and the spectrometer is ∼0.02 nm and ∼1.5 nm, respectively. The detection efficiency in the range of 520-600 nm is approximately the same as that in 740-820 nm. The transmission spectrum is recorded by using a photodetector (PD) and a data acquisition (DAQ) system triggered with laser wavelength sweeping. The laser power is kept less than 10 µW to avoid thermal broadening of a resonance for the transmission measurement. The spectral mapping is recorded with a spectrometer while slowly scanning the pump laser wavelength.

Effective interaction length between cavity modes and 2D materials

Because the maximum width of a uniform flake obtained by mechanical exfoliation is typically a few tens of micrometers, the interaction length per roundtrip is approximately same as the flake size across the equator of a microsphere. Ultrahigh-Q properties of our devices, nevertheless, significantly enhance the interaction since the input light circulates N times before the decay, where N is simply determined by the cavity Q and the roundtrip length (N reaches up to 3400 when the Q is 5×10^6 and the cavity radius is 40 µm). This effect allows to elevate the effective interaction length to 3.4–6.8 mm even in the flake size of up to 10–20 µm.

Theoretical estimation of threshold power for third-order nonlinear processes

Threshold pump power for parametric FWM and Raman oscillation is obtained by taking into account the power build-up factor in a microcavity coupled to an external waveguide, respectively [5, 6],

$$P_{\rm in}^{\rm FWM} = \frac{\omega n^2 V_{\rm eff}}{8Q^2 n_2 c\eta}, \tag{S2}$$

$$P_{\rm in}^{\rm SRS} = \frac{\omega^2 n^2 V_{\rm eff}}{4Q^2 c^2 \eta g_R}, \tag{S3}$$

where n is the refractive index, $V_{\rm eff}$ is the effective mode volume, n_2 is the nonlinear refractive index, c is the speed of light in a vacuum, η is the coupling efficiency to the waveguide ($\eta = 0.5$ is the critical coupling condition), and $g_R = 6.2\times10^{-14}$ is the Raman gain coefficient of silica. For a silica microsphere with a diameter of 80 µm, the parameters are assumed to be $n = 1.44$, $n_2 = 2.2\times10^{-20}$, and $V_{\rm eff} = 3287$ µm^3. The effective mode volume is calculated by FEM simulation.

Dynamic resonant phase-matching processes

Efficient nonlinear optical processes are achieved by dynamically obtaining resonant phase-matching from thermal and Kerr nonlinearities. The phase-matching condition described here is different from the conventional phase-matching for frequency conversion processes in nonlinear crystals, which is usually satisfied by aligning the incident angle and polarization of the pump lasers. The situation becomes more complicated in a microcavity system due to resonance effects as well as inherent material and geometric cavity dispersion [7]. Mode mismatch at the SH frequency is induced by the dispersion which hinders perfectly resonant phase-matching condition. We note that fulfilling the phase-matching condition is not too difficult as there are sufficiently large number of modes available. In order to locate resonances with efficient frequency conversion, we carefully scan the pump wavelength while monitoring the visible spectrum with a thermoelectrically cooled charged coupled device (CCD) attached to a spectrometer. Continuous wavelength scan of the pump laser induces changes in the refractive index of the cavity via thermal and Kerr effects, thus giving rise to the redshift of the resonance modes. Since cavity modes with different resonant frequencies and spatial mode distribution experience different amounts of the redshift,

the phase-matching condition can be fulfilled at certain pump wavelengths where efficient frequency conversion would occur.

SH intensity dependence on the pump polarization

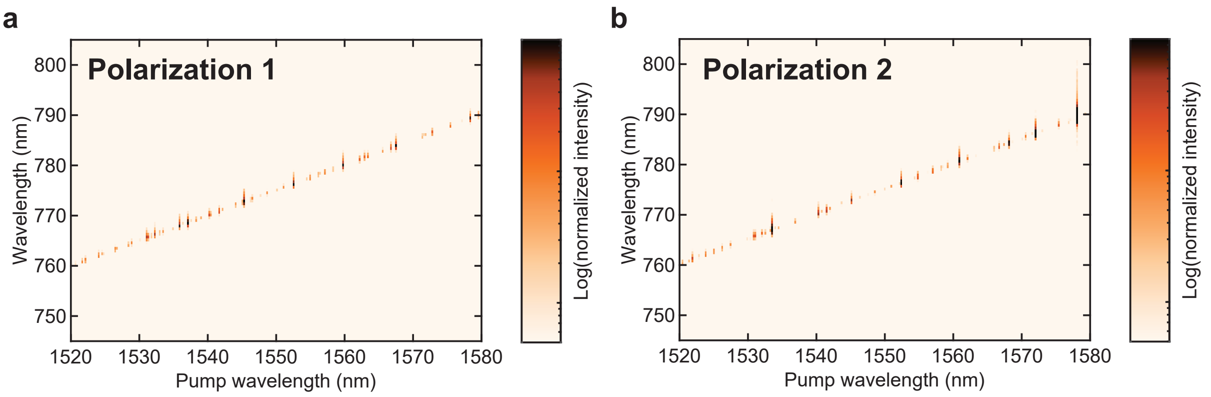

Supplementary Figure S4. Pump polarization dependence of SH light intensity. Spectral mapping of SH light intensity for two different pump polarizations (Polarization 1 and Polarization 2). The measurements are performed with a pump power of 3 mW.

The SH mapping for two different polarizations is shown in Fig. S4. We observe no significant dependence of SH intensity on the pump polarization even though the observed SH wavelengths clearly differ. The pump polarization is selected by using a fiber polarization controller before the excitation while monitoring the transmission spectrum. We note that the polarization of the SH light is not necessarily the same as the pump polarization because the SH light originated from TMD flakes exhibits a six-fold symmetric response (60° symmetry) [8] which allows coupling to either TE or TM modes.

SUPPLEMENTARY REFERENCES

[1] Haus, H. A. *Waves and fields in optoelectronics*; Prentice Hall, 1984.

[2] Kasumie, S.; Lei, F.; Ward, J. M.; Jiang, X.; Yang, L.; Nic Chormaic, S. Raman Laser Switching Induced by Cascaded Light Scattering. *Laser Photonics Rev.* **2019**, *13*, 1900138.

[3] Humphrey, M. J.; Dale, E.; Rosenberger, A.; Bandy, D. Calculation of optimal fiber radius and

whispering-gallery mode spectra for a fiber-coupled microsphere. *Opt. Commun.* **2007**, *271*, 124–131.

[4] Fujii, S.; Wada, K.; Sugano, R.; Kumazaki, H.; Kogure, S.; Kato, Y. K.; Tanabe, T. Versatile tuning of Kerr soliton microcombs in crystalline microresonators. *Commun. Phys.* **2023**, *6*, 1.

[5] Kippenberg, T. J.; Spillane, S. M.; Vahala, K. J. Kerr-Nonlinearity Optical Parametric Oscillation in an Ultrahigh-Q Toroid Microcavity. *Phys. Rev. Lett.* **2004**, *93*, 083904.

[6] Kippenberg, T. J.; Spillane, S. M.; Min, B.; Vahala, K. J. Theoretical and experimental study of stimulated and cascaded Raman scattering in ultrahigh-Q optical microcavities. *IEEE J. Sel. Topics Quantum Electron.* **2004**, *10*, 1219–1228.

[7] Fujii, S.; Tanabe, T. Dispersion engineering and measurement of whispering gallery mode microresonator for Kerr frequency comb generation. *Nanophotonics* **2020**, *9*, 1087–1104.

[8] Li, Y.; Rao, Y.; Mak, K. F.; You, Y.; Wang, S.; Dean, C. R.; Heinz, T. F. Probing Symmetry Properties of Few-Layer MoS_2 and h-BN by Optical Second-Harmonic Generation. *Nano Lett.* **2013**, *13*, 3329–3333.
